# Immunomodulatory effect of ultrasound-guided cryoablation in early breast cancer: pilot study on blood and surgical samples

**DOI:** 10.1186/s41747-025-00655-1

**Published:** 2025-12-22

**Authors:** Federica Pediconi, Francesca Galati, Marianna Nuti, Veronica Rizzo, Andrea Botticelli, Lucrezia Tuosto, Angelica Pace, Aurelia Rughetti, Giulia d’Amati, Bruna Cerbelli, Chiara Napoletano

**Affiliations:** 1 Department of Radiological, Oncological, and Pathological Sciences, Sapienza University of Rome, Rome, Italy; 2 Laboratory of Tumor Immunology and Cell Therapies, Department of Experimental Medicine, Sapienza University of Rome, Rome, Italy

**Keywords:** Breast neoplasms, Cryoablation, HMGB1 protein, Minimally invasive procedures, Regulatory, T-lymphocytes

## Abstract

**Objective:**

Cryoablation, a minimally invasive, image-guided procedure, induces tumor necrosis through freezing/thawing cycles. This pilot study investigates the immunomodulatory effects of cryoablation in early breast cancer (BC) patients by analyzing blood and surgical samples, with a focus on T-cell subsets, regulatory T cells (Tregs), serum cytokines, and high-mobility group box 1 (HMGB1) levels.

**Materials and methods:**

Ten patients with early BC (cT1 cN0) underwent ultrasound-guided cryoablation using a cryoablation system followed by surgical resection. Peripheral blood mononuclear cells were isolated at four time points: pre-ablation (T0), day 2–3 (T1), 2–3 weeks post-ablation (T2), and post-surgery (T3). Immune cell populations, including Tregs and activated CD137^+^ T cells, were analyzed via flow cytometry. Serum HMGB1 and cytokines (*e.g*., IL-1β, IL-6, and TNF-α) were measured using Luminex assays. The histopathological analysis assessed the tumor response to ablation and immune infiltrates.

**Results:**

Cryoablation significantly increased circulating HMGB1 levels at T1 (*p* = 0.047), with further elevation post-surgery (*p* = 0.023), suggesting immune activation. CD137^+^ T cells, predominantly the CD4^+^ subset, decreased significantly after surgery (*p* = 0.025), correlating with reduced interleukin-4 levels. Proliferating Tregs (Ki67^+^) also declined after the combined treatment (*p* = 0.046). Histopathology confirmed complete tumor ablation in 9 of 10 cases, with immune infiltrates, predominantly CD3^+^ lymphocytes (CD4^+^ and CD8^+^ equally represented).

**Conclusion:**

Cryoablation induces significant immunological changes, including the release of HMGB1, modulation of CD137^+^ T cells, and decreased Treg proliferation, highlighting its potential as both local and systemic immunomodulatory therapy.

**Relevance statement:**

Cryoablation triggers immune activation in early BC, as indicated by increased CD137^+^ T cells, reduced Tregs, elevated HMGB1, enhanced inflammatory cytokine release, and the presence of mild to intense inflammatory infiltrates in surgical samples. These findings suggest the potential efficacy of cryoablation in supporting immunotherapies in the treatment of BC.

**Key Points:**

- Cryoablation is a promising nonsurgical treatment for early-stage BC.
- The procedure may induce immune activation by increasing HMGB1 and modulating T-cell populations.
- Tregs appear to decrease after cryoablation, suggesting immunomodulatory potential.
- Histopathology confirms effective tumor ablation in most treated patients.
- Cryoablation shows immunomodulatory effects and may provide a rationale for future combination with immunotherapy.

**Graphical Abstract:**

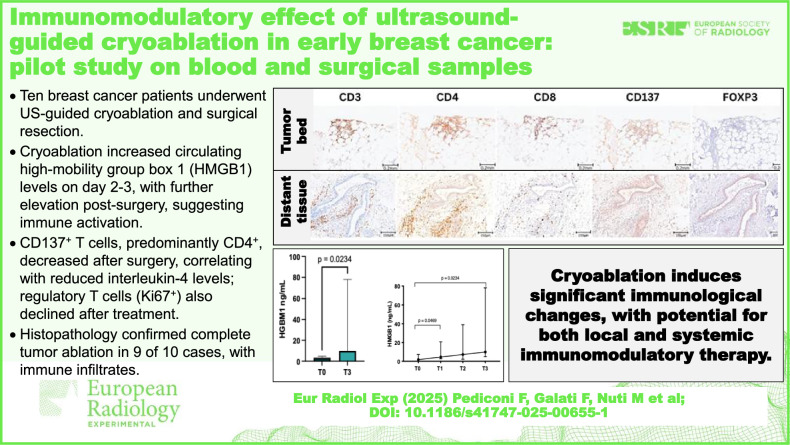

## Background

Breast cancer (BC) is the most common malignancy in women. In recent years, a steady increase in the incidence of early BC has been observed, due to continued improvements in the accuracy of screening programs [1]. Accordingly, several minimally invasive, image-guided treatment approaches are being investigated as alternatives to surgery, aiming to achieve oncologic outcomes equivalent to breast-conserving surgery while minimizing surgical risks. These treatments include radiofrequency, microwave, and laser ablation, cryoablation, and high-intensity focused ultrasound [2].

Cryoablation is a minimally invasive procedure that uses freezing and thawing cycles to induce targeted tissue necrosis while preserving the surrounding tissue [2]. A growing body of evidence indicates that cryoablation elicits localized tissue necrosis while modulating the immune system and promoting the release of inflammatory cytokines. Unlike thermal ablation techniques using hyperthermia [3, 4], cryoablation preserves the structural integrity of tumor-associated antigens and epitopes, which may serve as immune targets to trigger local and systemic immune responses [5–9]. Moreover, cell damage promotes the release or exposure of damage-associated molecular patterns (DAMPs), which alert the immune system to the presence of injured or dying cells, thereby triggering immune activation. High-mobility group 1 (HMGB1) is one such molecule, involved in the activation of innate immunity, particularly through the stimulation of dendritic cells via Toll-receptor 4 (TLR4) or advanced glycosylation end-product receptor (AGER) [10]. These interactions promote dendritic cell maturation, which successively activates a specific anti-tumor T-cell response [10]. Taken together, these findings suggest that cryoablation may exert beneficial immunomodulatory effects and has the potential to elicit a durable anti-tumor immune response.

CD137^+^ T cells have gained increasing attention in tumor immunology as a biomarker of antigen-specific T-cell activation. In several solid tumors, we have demonstrated the predictive and prognostic value of these cells [11, 12]. CD137 molecule is a tumor necrosis factor receptor−TNFR family member expressed by CD4 and CD8 lymphocytes after activation. Its interaction with CD137L triggers anti-apoptotic signals, increases T cell functions, and the transcription of CD8 main genes [13]. Moreover, this receptor has also been used as a biomarker of selection for isolating tumor-reactive T cells from the tissue, highlighting its relevance in isolating antigen-specific T cells with tumor reactivity [14].

In contrast, regulatory T cells (Tregs) represent one of the main lymphocyte subsets with immunosuppressive activity. Forkhead box protein P3 (FOXP3) is the master regulator of this population, and high expression levels of that molecule in tumor tissue indicate a highly immunosuppressive tumor milieu. The role of high levels of Tregs in blood samples is controversial and strictly correlated with tumor histotype. In hormone receptor-positive, human epidermal growth factor receptor 2 (HER2)-negative BC patients, we observed a marked reduction in circulating Tregs following CDK4/6 inhibitor treatment in responders [11]. In naïve non-small cell lung cancer patients, high levels of Tregs at baseline identify patients with longer survival [12].

In this pilot study, we aimed to investigate the immunomodulatory effects of cryoablation on blood and surgical samples, focusing on CD3^+^, CD4^+^, and CD8^+^ T cells, as well as key subsets involved in anti-tumor activity (CD137^+^) and immune suppression (Tregs). In parallel, we studied changes in serum levels of the inflammation molecule HMGB1 and cytokines.

## Materials and methods

### Study design

The study was approved by the Institutional Review Board of “Sapienza” University of Rome (Ref. 6528, approved 24.11.2021) (clinicaltrials.gov/study/NCT05727813). This was a prospective pilot study involving patients with early-stage BC (cT1 cN0) who underwent ultrasound-guided cryoablation prior to planned breast surgery, with a cryo-feasible cancer location.

### Patient eligibility

Patients > 18 years of age with a single, biopsy-proven, early-stage invasive BC up to 20 mm in diameter (cT1 cN0), scheduled for breast surgery (mastectomy or lumpectomy), not eligible for systemic neoadjuvant therapy, were included. Lesions had to be clearly visible on ultrasound, with a minimum distance of 1.5 cm from the skin and 2 cm from the nipple. Exclusion criteria were histological diagnosis of ductal carcinoma *in situ* (DCIS); lesions with microcalcifications as the only evidence of BC; previous history of BC; the presence of breast implants; inability to undergo cryoablation treatment; pregnancy; breastfeeding; or recent childbirth.

### Cryoablation

All cryoablation procedures were conducted by a radiologist with 20 years of experience in breast interventional radiology (F.P.) using the ICEfx Cryoablation System (Boston Scientific). This system featured a 17-gauge disposable cryoprobe (ICE SPHERE 1.5, Boston Scientific), capable of generating an ice ball with a maximum cross-sectional diameter of 33 mm (short axis) × 37 mm (long axis). The system operated using argon as the cryogen and included a resistance heater for the thawing process. The cryoablation procedure lasted approximately 25 min, consisting of an initial freezing phase (10 min), a thawing phase (5 min), and a second freezing phase (10 min). In all cases, a single cryoprobe was inserted through a small skin incision under local anesthesia (1% lidocaine) without the need for patient sedation [15].

### Surgery

Preoperative axillary lymph node status was always assessed by ultrasound. All patients underwent surgical resection of the primary tumor and axillary sentinel lymph node excision within 21 days after cryoablation, with up to four lymph nodes removed to ensure oncologic safety and achieve an adequate aesthetic result. All patients also underwent preoperative wire-guided localization of the BC lesion.

### Peripheral blood mononuclear cells and serum collection

Peripheral blood mononuclear cells were isolated from patients with a confirmed diagnosis of BC using Ficoll Hypaque (Lympholite-H) before cryoablation (T0), 2–3 days and 2–3 weeks after cryoablation (T1 and T2, respectively), and after surgery (T3). Concurrently, patients’ sera were collected using BD Vacutainer Plus Plastic Serum tubes (Becton Dickinson) after centrifugation at 1800 rpm for 10 min. Samples were cryopreserved (-80 °C) until use.

### HMGB1 detection

HMGB1 concentration was detected in patients’ sera using an enzyme-linked immunosorbent assay (ELISA) kit (TECAN, Zürich, Switzerland) according to the manufacturer’s protocol.

### Immunophenotyping

Immunophenotyping was performed by flow cytometry using a multiparametric analysis combining different conjugated anti-human monoclonal antibodies. Tregs were analyzed using anti-KI67 (B6818 clone), CD137 (4B4-1 clone), PD1 (PD1.3 clone), CD25 (B1.49.9 clone), FOXP3 (259D clone), CD45RA (2H4 clone), CD4 (13B8.2 clone), and CD3 (UCHT1 clone) antibodies (all from Beckman Coulter, Brea, CA). T cells were characterized by anti-CD137 (4B4-1 clone), PD1(PD1.3 clone), CCR7 (G043H7 clone), CD28 (CD28.2 clone), CD45RA (2H4 clone), CD8 (B9.11 clone) CD3 (UCHT1 clone) (all from Beckman Coulter, Brea, CA, USA). Live cells were identified using LIVE/DEAD fixable yellow dead cell staining kit (Invitrogen, Waltham, MA, USA). FOXP3/Transcription Factor Staining Buffer Set (Invitrogen) was used for intracellular staining following the manufacturer’s instructions. Tregs were evaluated by gating on FSC-A/SSC-A, SSC-A/CD3^+^, live cells/CD3^+^, and SSC-A/CD4^+^ cells. Twenty thousand SSC-A/CD4^+^ cells were acquired. Treg subpopulations were studied using the CD45RA marker as active (CD4^+^CD25^high^FOXP3^high^CD45RA^-^), resting (CD4^+^CD25^+^FOXP3^low^CD45RA^+^), and nonsuppressive (CD4^+^CD25^low^FOXP3^low^CD45RA^-^) Tregs. For the analysis of active T cells (CD137^+^ and PD1^+^), lymphocytes were selected by gating on FSC-A/SSC-A, SSC-A/CD3^+^, and live cells/CD3^+^. Ten thousand live cells/CD3^+^ events were acquired and analyzed. Samples were acquired with DxFLEX Flow Cytometer (Beckman Coulter) and analyzed by FlowJo software (version 10.8.8, Becton Dickinson).

### Circulating molecules

Cytokines and immune checkpoints were detected in patients’ sera using the Inflammation 20-Plex Human ProcartaPlex and Immuno-Oncology Checkpoint 14-Plex Human ProcartaPlex Panels (ThermoFisher Scientific).

The first panel included E-Selectin, GM-CSF, ICAM/CD54, IFNα, IFNγ, IL1α, IL1β, IL4, IL6, IL8, IL10, IL12p70, IL13, IL17A/CTLA8, IP10/CXCL10, MCP1/CCL2, MIP1α/CCL3, MIP1β/CCL4, P-Selectin, TNFα. The second panel detected BTLA, GITR, HVEM, IDO, CD137, CD27, CD152, LAG-3, PD-1, PD-L1, PD-L2, TIM-3, CD28, and CD80 as immune checkpoints. Molecules were evaluated by Luminex multiple assays and analyzed using Bioplex Manager MP software (Bio-Rad).

### Pathological analysis and immunophenotyping

The pathological response to cryoablation was assessed on surgical samples by two breast pathologists (B.C. and G.d.A.), each with more than 15 years of experience in breast pathology. All specimens were measured and weighed. The treated area was identified on the cut surface by visible hemorrhage and steatonecrosis. The entire cryoablated area and the surrounding breast tissue were sampled for histology. Serial sections were cut from each paraffin block, stained with hematoxylin–eosin, and observed under light microscopy to evaluate the degree of pathological response and the histologic features of the tumor bed, including the presence, extent, and distribution of immune cells. The degree of inflammatory infiltrates was semiquantitatively graded as mild (less than two foci at 10× magnification) and intense (more than two foci at 10× magnification).

Immunophenotyping of the inflammatory infiltrates was performed with CD3 (LN10, RTU, Leica-Biosystem, Nussloch, Germany); CD4 (4B12, RTU, Leica-Biosystem), CD8 (4B11, RTU, Leica-Biosystem), CD137 (AB197942, 1:100, Abcam); FOXP3 (AB20034, 1:100, Abcam) using an automated immunostainer (Leica-Bond Max Leica Microsystems GmbH) with the Bond Polymer Refine Detection kit (Leica-Bond Max) according to manufacturer’s instructions.

### Statistical analysis

Data for baseline patient and lesion characteristics are presented as median and range (min–max). Data for immunological analyses are presented as median and interquartile range (IQR). Descriptive statistics were performed with GraphPad Prism version 10 (GraphPad Software, Inc.). The Wilcoxon matched-pairs signed rank test was used to analyze groups and compare time points. A *p*-value < 0.05 was considered statistically significant. Some analyses were exploratory in nature and not powered for statistical significance; these results were interpreted as hypothesis-generating.

## Results

### Study population characteristics

From July 2022 to January 2023, 10 patients with a biopsy-proven early BC, < 20 mm in size, were included in this pilot study. All patients included in the study underwent cryoablation. The patient population was the same as described in our previous publication [15]. The median lesion size on imaging was 10 mm (range 6–16 mm).

The median age of the patients was 64.5 years (range 47–82), 9 out of 10 patients were menopausal, and none of the patients had a family history of malignant breast disease. Of the 10 patients enrolled, 8 had a biopsy-proven diagnosis of invasive no special type BC, and 2 had invasive lobular carcinoma; 8 of 10 were of intermediate nuclear grade. Four lesions were classified as luminal A-like, 5 as luminal B-like, and 1 as HER2-enriched.

### Surgical procedure

All 10 patients underwent surgical resection within 21 days of cryoablation. Surgery consisted of quadrantectomy and sentinel lymph node biopsy in all 10 cases. The specimen size ranged from 6 × 4 × 2 cm to 9 × 8.5 × 3 cm, and the median weight of the samples was 40.5 g (range 15–110 g).

### Immunomodulatory effects observed after cryoablation and surgery

### HMGB1 progressively increased after cryoablation and surgery

**Fig. 1:**
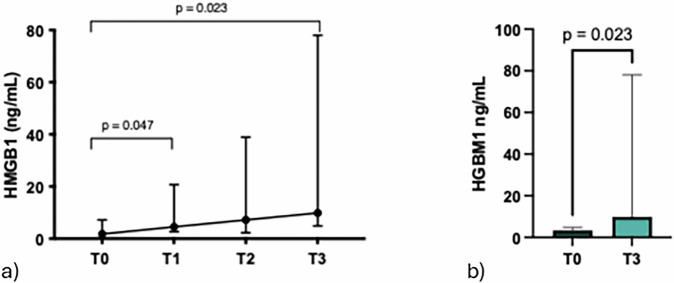
The inflammatory/damage molecule HMGB1 is progressively released in the serum of cancer patients after cryoablation (T0-T1, *p* = 0.047) until surgical resection of the primary tumor (T0-T3, *p* = 0.023). **a** The connecting line represents the trend of HMBG1 level before (T0) and after (T1) cryoablation and before (T2) and after (T3) surgery. **b** Histograms show the median concentration of HMGB1 (IQR) evaluated in ten BC patients at baseline (T0) and after the cryosurgery procedure (T3). HMGB1 increased significantly from T0 to T1 (*p* = 0.047) and from T0 to T3 (*p* = 0.023). HMGB1, High-mobility group box 1; IQR, Interquartile range

Patient’s sera were analyzed for the release of HMGB1, a damage molecule, after cryoablation and surgery (Fig. 1). The circulating levels of HMGB1 significantly increased from T0 to T1 (*p* = 0.047). These levels remained elevated before surgery (T2) and further increased following surgery compared to baseline (T3; *p* = 0.023).

### Cryoablation followed by surgery decreased the CD4^+^CD137^+^ T cell subset and the serum level of IL-4

**Fig. 2:**
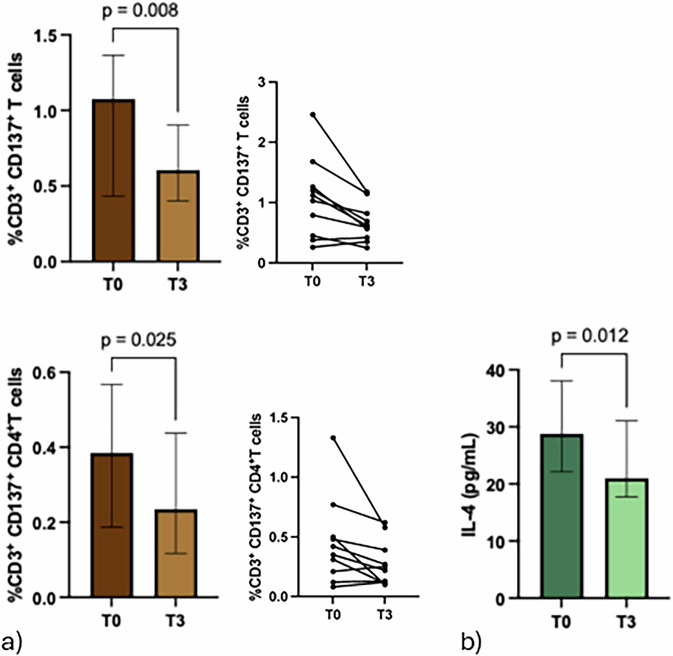
Cryoablation followed by surgery induced a significant decrease of CD137^+^ T cell subsets (total and CD4^+^), and IL-4. **a** Histograms show the median percentage of CD137⁺ T cell subsets (total and CD4⁺) (IQR) evaluated in ten BC patients at baseline (T0, grey histograms) and after cryoablation followed by surgery (T3, light grey histograms). Symbol-and-line graphs represent the percentage of CD137⁺ T cell subsets (total and CD4⁺) for each patient before cryoablation and after surgery. **b** Histograms show the median concentration of IL-4 (pg/mL; IQR) evaluated by Luminex assay at T0 and T3. Total CD137⁺ T cells (*p* = 0.008) and CD4⁺CD137⁺ T cells (*p* = 0.025) decreased significantly from T0 to T3, while IL-4 levels also significantly decreased (*p* = 0.012). IL-4, Interleukin 4; IQR, Interquartile range

Several circulating lymphocyte populations (total CD3, CD8, CD4, naïve, effectors, central memory, effectors memory, CD137, and PD1) were evaluated in the 10 BC patients at different time points. Results showed a significant decrease in the activated CD137^+^ T cells from T0 to the end of the entire procedure (T3) (*p* = 0.008) (Fig. 2a), while no differences were observed after cryoablation (T1, data not shown). The decrease observed between T0 and T3 was mainly ascribed to CD4^+^CD137^+^ T cells (*p* = 0.025) compared to the cytotoxic CD137 T cell subset. Concurrently, a reduction in the levels of the proinflammatory cytokine IL-4 was observed from T0 to T3 (Fig. 2b). Moreover, several other cytokines and soluble immune checkpoints were evaluated in the sera of the patient. No significant differences were observed after cryoablation or tumor surgical resection.

### Proliferating Tregs decreased after cryoablation followed by surgery

**Fig. 3:**
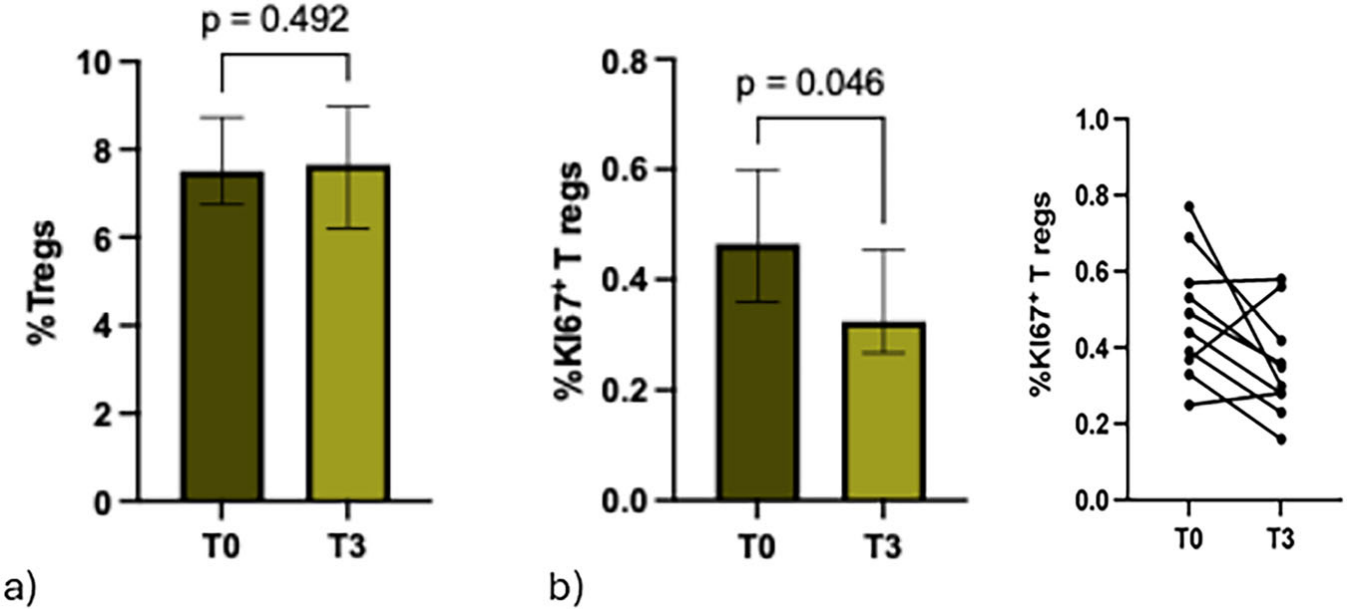
The cryoablation followed by surgery significantly decreased proliferative Tregs. **a** Histograms represent the median percentage of total Tregs (IQR) and (**b**) proliferative Tregs (Ki67⁺ Tregs) (IQR) evaluated in ten BC patients at baseline (T0, grey histograms) and after cryoablation followed by surgery (T3, light grey histograms). Symbol-and-line graphs show the percentage of Ki67⁺ Tregs at T0 and T3. Proliferating Ki67⁺ Tregs significantly decreased from T0 to T3 (*p* = 0.046). Tregs, Regulatory T cells; IQR, Interquartile range

Circulating immunosuppressive Tregs were analyzed in the ten BC patients from T0 to T3. The levels of total Tregs remained unchanged throughout the entire procedure (Fig. 3a), as did the resting and non-suppressive Treg subsets (Supplementary Fig. S1). A decreasing trend was observed in the activated Tregs, although it did not reach statistical significance (*p* = 0.074). When the levels of proliferating Tregs were assessed, a significant decrease in Ki67^+^ Tregs was observed (*p* = 0.046) from baseline to T3 (Fig. 3b).

### Histopathological analysis

In all cases, histopathologic analysis of the surgical specimens confirmed the presence of a central hemorrhagic area, surrounded by steatonecrosis. Tumor ablation was complete (ypT0) in 9 out of 10 patients, with residual invasive carcinoma detected only in one case, located at the edge of the treatment zone, likely due to targeting failure. However, in two of the nine ypT0 cases, microscopic examination of the tissue outside the cryoablated area revealed small foci of intermediate-grade DCIS. Therefore, a complete pathological response, defined as the absence of both invasive and *in situ* carcinoma, was achieved in 7 out of 10 patients.

**Fig. 4:**
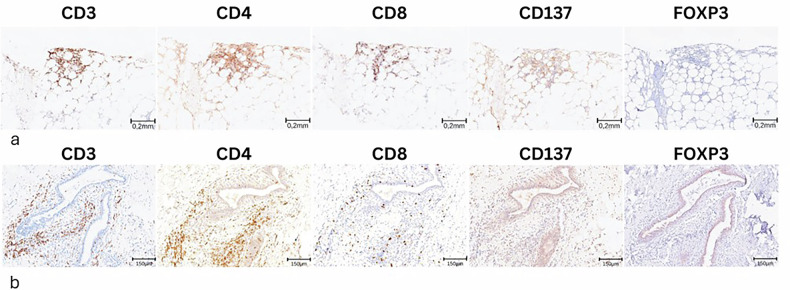
Immunohistochemical evaluation of immune infiltrates on surgical samples. **a** Representative images of immune infiltrates observed in the tumor bed and (**b**) in normal breast tissue outside the cryoablated area. Immunohistochemistry highlighted in both cases the prevalence of CD3^+^ lymphocytes, with CD4^+^ and CD8^+^ sub-populations equally represented. CD137^+^ immune cells were present at low levels, and no FOXP3^+^ cells were detected. FOXP3, Forkhead box P3

The presence of immune infiltrates was observed in all samples and was scored as mild in 4 and intense in the remaining 6 cases. Most inflammatory cells were CD3^+^ lymphocytes, with CD8^+^ and CD4^+^ sub-populations equally represented. CD137^+^ cells were detectable in 7/10 cases, although in low amounts. No FOXP3+ elements were observed. Immune infiltrates were detected both in the tumor bed (Fig. 4a) and in normal breast tissue beyond the cryoablation area (Fig. 4b), where inflammatory infiltrates surrounded normal ducts, the foci of lobular carcinoma *in situ*, and the small foci of DCIS.

## Discussion

Cryoablation has recently emerged as a potential alternative to surgery for selected BC patients who are ineligible for surgical resection. Although BC cryoablation is still considered in the experimental phase, complete tumor cell death within the ablated zone has been observed in most cases [2–4, 16].

One of the most intriguing aspects of cryoablation is its potential for immune activation. The concept of the cryo-immune response is based on anecdotal reports of distant tumor regression following cryoablation of various primary tumors, including BC [6–8]. Theoretically, *in situ* cryoablation is ideal for generating an anti-tumor immune response due to the mechanism by which cryoablation induces the release of tumor-associated antigens and several other molecules that can modulate the immune response [5–8].

In this study, we aimed to explore the potential of cryoablation for treating early-stage BC and its effect on the modulation of the immune system. The findings provide significant insights into the role of cryoablation as a less invasive alternative to surgery and a potential inducer of an immune response against the tumor.

We observed a significant increase in serum levels of HMGB1, a protein associated with tissue damage and the innate immune response that can interact with immune cells and trigger inflammatory responses, potentially promoting antitumor immunity [10, 17]. This suggests that cryoablation could potentially activate the immune system by releasing this molecule, which could, in turn, stimulate an antitumor immune response. The increase in HMGB1 after both cryoablation and surgery supports the hypothesis that these combined procedures have the potential to enhance the immunogenicity of the treatment. The increase in inflammatory status, combined with the release of tumor-associated antigens induced by the treatment, implies the activation of CD137^+^ T cells, particularly CD4^+^CD137^+^ T cells, which secrete the pro-inflammatory cytokine interleukin 4 (IL-4).

Concurrently, a trend towards an increase in proliferating T cells, and particularly CD8 lymphocytes in blood, further suggests a modulation of the immune response (Supplementary Fig. S1b). This activation is further supported by the analysis of steatonecrosis nodules, where the presence of high levels of lymphocytes (CD4^+^ and CD8^+^) and CD137^+^ cells, along with the absence of Tregs (FOXP3^+^ cells), raises the hypothesis that cryoablation may modulate the immune activation/suppression balance toward a more activated immune state. This finding is consistent with previous evidence of FOXP3 downregulation and Treg reduction after cryoablation [18]. These data are especially relevant because the outer zone of cryoablation undergoes apoptosis, typically considered immunosuppressive. The interplay between necrosis and apoptosis is crucial in defining the type of immune response evoked [13].

After surgery, the analysis of circulating T cells revealed a significant decrease in CD4^+^CD137^+^ T cells and a reduction in IL-4 levels. This reduction may reflect the resolution of an immune activation, suggesting a self-limiting regulatory phase following initial stimulation. Indeed, a concurrent decrease in proliferating Tregs after surgery is observed in blood samples, confirming the extinguishment of the systemic inflammatory response. In line with our results, several preclinical studies on different tumor types highlighted cryoablation’s capacity to modulate the immune response, which further enhances anti-tumor immunity [19, 20]. In colon and lung carcinoma, cryoablation increases tumor-infiltrating CD8 T cells while decreasing Treg levels [16, 18]. Similar results have been observed in breast carcinoma, where a local increase in cytotoxic T cells occurs alongside a reduction in Tregs and tumor expression of programmed death-ligand 1 (PD-L1).

Furthermore, as we have noted, recent research supports cryoablation’s potential not only as a localized treatment but also as a procedure to induce systemic anti-tumor immunity [7]. This evidence has drawn more attention to cryoablation as a potential therapy to combine with immunotherapy, suggesting a synergistic effect between these therapies for improving clinical outcomes in patients [9, 14]. Indeed, several data demonstrate that cryoablation plus immunotherapy can exert anti-tumor effects on both primary tumors and distant metastases [9], confirming that the synergy of different therapeutic strategies initially priming the immune system (cryoablation) and then expanding and reinforcing the pre-existing immune response (immune checkpoint inhibitors) could imply a clinical disease control, offering real and prolonged benefits for cancer patients [15]. This innovative therapeutic strategy, by enhancing the immune system, prepares cancer patients to receive hormone-based therapy, which typically follows surgery. Results from the ICE 3 trial further supported this hypothesis. One hundred ninety-six women aged over 60 years with early-stage hormone receptor-positive HER2-enriched BC patients who received cryoablation followed by endocrine therapy had an ipsilateral breast tumor recurrence rate of 3.7% at 5 years, compared to an IBTR rate of 4. 3% in women who underwent surgery [21]. While the ICE3 results support the oncologic safety of cryoablation, their protocol differed from ours, as ICE3 combined cryoablation with endocrine therapy, whereas in our study, it was followed by surgical resection.

From a histopathological perspective, tumor ablation was complete in 9 out of 10 patients, confirming the effectiveness of cryoablation in destroying tumor tissue. The presence of residual invasive tumor in one case, located at the edge of the cryo-treated area, suggests the need for careful probe positioning to avoid marginal undertreatment. Our 90% ablation rate aligns with the range reported in the literature [21–23] and provides encouraging support for the potential oncological safety of this procedure, especially when considering that in selected populations, such as those included in the ICE3 trial, cryoablation alone has led to comparable recurrence rates to surgery. Although our study was not designed to assess long-term outcomes, these preliminary results indicate that, in well-selected patients, cryoablation may offer a reliable alternative to conventional surgery.

An interesting aspect that emerged from our analysis is the presence of immune infiltrates in both the tumor bed and the surrounding normal breast tissue, indicating a widespread immune response not confined to the treated area. This phenomenon could have implications for the long-term efficacy of cryoablation, suggesting that immune activation might play a role in preventing local and possibly in-breast distant recurrences.

In conclusion, this pilot study supports the potential of cryoablation followed by surgical removal as a novel therapeutic approach for treating early-stage BC. Its effectiveness in destroying the tumor and its ability to modulate the immune response make it a promising option. These preliminary results, along with the existing literature, pave the way for further studies to explore the combination of cryoablation with other forms of immunotherapy, such as immune checkpoint inhibitors, to enhance clinical outcomes for BC patients. Further investigation into these approaches may inform future clinical strategies and potentially improve outcomes in BC management.

## Supplementary information

**Additional Supplementary Fig. S1.** Treg subsets and proliferating T cells. (a) Median percentages of non-suppressive (CD45RA⁻FOXP3^low^), resting (CD45RA⁺FOXP3⁺), and activated (CD45RA⁻FOXP3^hi^) Tregs within CD3⁺CD4⁺CD25⁺ cells are presented as median % (IQR) at baseline (T0) and after surgery (T3). No significant differences were observed, with a trend toward reduction in activated Tregs. (b) Median percentages of proliferating T cells (CD3⁺Ki67⁺) and cytotoxic T cells (CD8⁺Ki67⁺) are presented as median % (IQR) at T0, T2 (2–3 weeks post-cryoablation), and T3. FOXP3 Forkhead box P3, Tregs regulatory T cells, IQR interquartile range.

## Data Availability

The datasets used and analyzed during the current study are available from the corresponding author upon reasonable request.

## Abbreviations

BC: Breast cancer
DCIS: Ductal carcinoma *in situ*
FOXP3: Forkhead box protein P3
HER2: Human epidermal growth factor receptor 2
HMGB1: High-mobility group box 1
PD-L1: Programmed death-ligand 1
Tregs: Regulatory T cells
