# Supplementary material for: Immunomodulatory effect of ultrasound-guided cryoablation in early breast cancer: pilot study on blood and surgical samples
Source: Eur Radiol Exp. 2025 Dec 22;9:124. doi: 10.1186/s41747-025-00655-1 (PMC12722633; doi:10.1186/s41747-025-00655-1)
Supplement: Supplementary file 1 — Additional Supplementary Fig. S1. Treg subsets and proliferating T cells. (a) Median percentages of non-suppressive (CD45RA⁻FOXP3^low^), resting (CD45RA⁺FOXP3⁺), and activated (CD45RA⁻FOXP3^hi^) Tregs within CD3⁺CD4⁺CD25⁺ cells are presented as median % (IQR) at baseline (T0) and after surgery (T3). No significant differences were observed, with a trend toward reduction in activated Tregs. (b) Median percentages of proliferating T cells (CD3⁺Ki67⁺) and cytotoxic T cells (CD8⁺Ki67⁺) are presented as median % (IQR) at T0, T2 (2–3 weeks post-cryoablation), and T3. FOXP3 Forkhead box P3, Tregs regulatory T cells, IQR interquartile range. [file 41747_2025_655_MOESM1_ESM.pdf]

Immunomodulatory Effect of Ultrasound-Guided Cryoablation in Early Breast Cancer: Pilot Study on blood and surgical samples

ELECTRONIC SUPPLEMENTARY

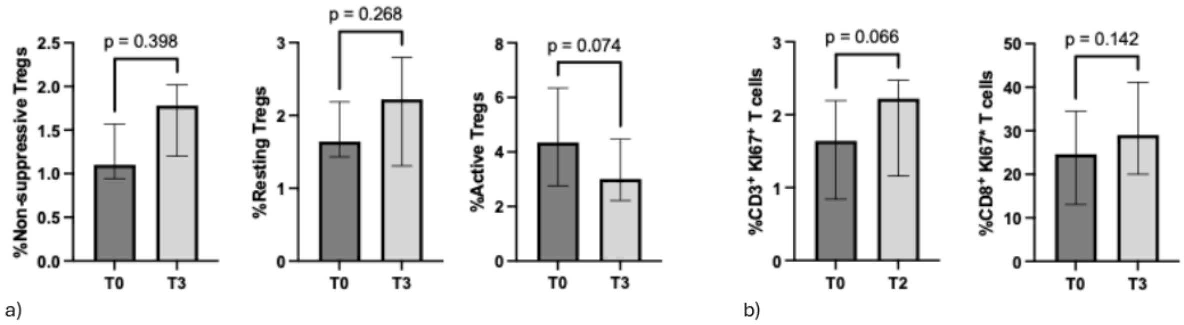

Fig. S1. Treg subsets and proliferating T cells.

(a) Median percentages of non-suppressive (CD45RA$^-$FOXP3^low^), resting (CD45RA$^+$FOXP3$^+$), and activated (CD45RA$^-$FOXP3^hi^) Tregs within CD3$^+$CD4$^+$CD25$^+$ cells are presented as median % (IQR) at baseline (T0) and after surgery (T3). No significant differences were observed, with a trend toward reduction in activated Tregs.

(b) Median percentages of proliferating T cells (CD3$^+$Ki67$^+$) and cytotoxic T cells (CD8$^+$Ki67$^+$) are presented as median % (IQR) at T0, T2 (2–3 weeks post-cryoablation), and T3.

FOXP3 Forkhead box P3, *Tregs* regulatory T cells, *IQR* interquartile range.
